# Design, Fabrication, and Characterization of a Novel Optical Six-Axis Distributed Force and Displacement Tactile Sensor for Dexterous Robotic Manipulation

**DOI:** 10.3390/s23249640

**Published:** 2023-12-05

**Authors:** Olivia Leslie, David Córdova Bulens, Stephen J. Redmond

**Affiliations:** School of Electrical and Electronic Engineering, University College Dublin (UCD), D04 V1W8 Dublin, Ireland

**Keywords:** force, torque, displacement, 3D, distributed, tactile, sensor, array, optical

## Abstract

Real-time multi-axis distributed tactile sensing is a critical capability if robots are to perform stable gripping and dexterous manipulation, as it provides crucial information about the sensor–object interface. In this paper, we present an optical-based six-axis tactile sensor designed in a fingertip shape for robotic dexterous manipulation. The distributed sensor can precisely estimate the local XYZ force and displacement at ten distinct locations and provide the global XYZ force and torque measurements. Its compact size, comparable to that of a human thumb, and minimal thickness allow seamless integration onto existing robotic fingers, eliminating the need for complex modifications to the gripper. The proposed sensor design uses a simple, low-cost fabrication method. Moreover, the optical transduction approach uses light angle and intensity sensing to infer force and displacement from deformations of the individual sensing units that form the overall sensor, providing distributed six-axis sensing. The local force precision at each sensing unit in the X, Y, and Z axes is 20.89 mN, 19.19 mN, and 43.22 mN, respectively, over a local force range of approximately ±1.5 N in X and Y and 0 to −2 N in Z. The local displacement precision in the X, Y, and Z axes is 56.70 μm, 50.18 μm, and 13.83 μm, respectively, over a local displacement range of ±2 mm in the XY directions and 0 to −1.5 mm in Z (i.e., compression). Additionally, the sensor can measure global torques, $T_{x}$, $T_{y}$, and $T_{z}$, with a precision of of 1.90 N-mm, 1.54 N-mm, and 1.26 N-mm, respectively. The fabricated design is showcased by integrating it with an OnRobot RG2 gripper and illustrating real-time measurements during in simple demonstration task, which generated changing global forces and torques.

## 1. Introduction

The human fingertip has been shown to have the highest density of mechanoreceptors in the human body, providing tactile feedback about deformations and slips occurring on the skin [1]. This feedback allows humans to perform complex dexterous manipulation with ease. Tactile sensors aim to provide similar feedback with direct measurements of the object/sensor interface during contact so that, when integrated into a robotic gripper, they can enable the robotic gripper to maintain a secure grasp [2]. To mimic human tactile feedback, tactile sensors require a spatially distributed array of sensing units [3,4], providing global and localized information about the contact. However, most tactile sensors to date possess a shape and size that makes it challenging to integrate them into existing robotic fingers/grippers due to the small space of most robotic fingers [5,6]. As a result, current robotic grippers and existing tactile sensors are incompatible, and most commercially available robotic grippers lack tactile sensation [5,7,8].

Among most tactile sensors described in the literature [3,9,10,11,12,13,14,15,16,17], only a few are easily integrated into existing robotic grippers. The companies Touchence, XELA Robotics, and Contactile have developed sensor arrays that can be easily added to robotic grippers, mainly due to their small size. Touchence [18] uses a microelectromechanical system (MEMS) piezoelectric method to make a small six-axis sensor measuring force, with their smallest version being 9 × 9 × 5 mm, which will fit on almost all robotic grippers. However, piezoelectric sensors tend to be fragile and are only suitable for dynamic measurements [19]. Contactile has a compact nine-pillar three-axis force sensor array, which has been effectively added to the fingertip of robotic grippers [20]. uSkin has a successful integration of their sensor with the Allegro and iCub hands, as the small size of the tactile sensor array allows the ability to cover fingers and whole hands effectively. Yet, in practice, magnetic-based sensors can potentially be affected by external magnetic sources [17,21]. However, more often, when requiring six-axis force/torque measurements in robotic gripping, rigid commercial force/torque sensors are added to grippers, for example the popular ATI Industrial Automation (Apex, NC, USA) force/torque sensor family [22]. However, these sensors are flat, rigid, and cylindrical (17 mm diameter for the smallest available sensor) and only return a single-point force/torque estimate. These also lack other properties that tactile sensors have from having a soft outer skin, such as conforming to the object’s shape, thus preventing damage and improving the grasp. Combined with the softness, tactile sensors have the ability to measure force over a spatially distributed area, allowing the whole contact interface to be assessed, meaning a pressure distribution can be determined, as well as slip events, which warns of the impending loss of the grip, showing tactile sensors to be essential for dexterous robotic manipulation [2].

Distributed tactile sensing from tactile sensors provides rich information about the contact between the robot finger and the object and more accurately mimics the human-fingertip-sensing mechanisms [2,8]. Indeed, previous studies showed that distributed tactile sensing can help improve robotic gripper performance, as it can continuously provide contact interface information, including the direction and magnitude of forces and torque. Having rich haptic information about the manipulated object [2] allows for the force feedback control needed for stable grasping [23]. However, most of the distributed sensing that has been implemented on robotic fingertips and/or hands tends to only have a one-axis force/pressure measurement per sensing unit; that is, they measure pressure. For instance, strain gauge pressure sensors are more easily integrated with robotic grippers [24], but do not provide six-axis force/torque information. Yoshikai et al. developed a triaxial strain gauge sensor for the skin of the Marca robot; however, the sensor’s sensing units have a proportionally large area, restricting a dense distribution of sensing units [25]. More recently, Kim et al. developed a strain gauge sensor to detect slip on a robotic hand, with a liquid metal strain gauge inside a silicone cone, which can detect forces in both the X and Y axes. However, the sensor’s overall size, including the casing, is 11.125 × 6 × 13 mm${}^{3}$ [26], meaning the sensor is still proportionally large compared to a robotic hand, especially considering this is not distributed sensing and only provides two axes of measurement [26]. Optical tactile sensing has been shown to be an effective method for providing multi-axis sensing and tactile perception [2,10,16,19].

### 1.1. Optical-Based Tactile Sensors

Optical tactile sensing utilizes the properties of light to transduce force, meaning that optical tactile sensors have an excellent resilience with respect to electromagnetic interference, have a fast response [6], and are becoming more popular due to the high spatial density/resolution achievable, especially for distributed sensing. We have broadly grouped optical tactile sensors into two groups based on whether they use a camera or not in their sensing method.

#### 1.1.1. Camera-Based Optical Tactile Sensors

Many successful camera-based sensors in the literature utilize markers on a sensor skin to estimate the skin deformation and infer tactile properties from this [6,10]. While the use of a camera allows for a very high spatial resolution, the camera also requires a larger sensor size to maintain the focus of the camera. This makes their integration into existing grippers challenging [12,27,28]. The GelSight family of sensors has an incredibly high spatial resolution, which allows them to obtain high-resolution tactile images of the 3D surface topography, and have been shown to be successful in many robotic applications [12]. However, the sensors have a large overall size, even the improved fingertip GelSight sensor, which is about half the size of the first GelSight sensor, still has a focal length of 35 mm, meaning the overall fingertip sensor remains bulky [29]. In recent years, there has been a trend to develop smaller versions of the camera-based sensors, with folded optics (i.e., mirrors) used to reduce the sensor’s dimensions. The GelSlim sensor utilizes this method to achieve a thickness of 20 mm [27]; however, this adds other complexities to the design and reduces the sensor’s precision [27]. A trade-off for the high spatial resolution is that camera-based sensors generally have a poor temporal resolution, which is limited by the frames per second (FPS) captured by the camera. This can result in high-frequency information being lost. Moreover, camera-based sensors, such as sensors in the GelSight family and the TacTip, have substantial data processing requirements due to the need to process the high-resolution video stream [10,30]. Reducing the size of camera-based optical tactile sensors allows them to be integrated with robotic grippers more easily, but this comes at the cost of the accuracy of force estimates as the focal length required by the camera to obtain a clear image makes camera-based sensor fundamentally large.

#### 1.1.2. Non-Camera-Based Optical Tactile Sensors

Non-camera-based tactile sensors typically utilize changes in light intensity to infer tactile information. The use of photodetectors has been successful at providing precise measurements of deformation and force at a point on the external surface of the sensor skin. Eliminating the use of a camera inherently makes the sensor more compact and easier to integrate with existing robotic grippers. Therefore, non-camera-based optical tactile sensors present the potential of a small thickness, low processing requirements, high temporal resolution, and the ability to cover non-planar surfaces.

For instance, optical fiber tactile sensors have a small thickness (<3 mm), with the ability to measure pressure, vibration, and 3D force [31,32], and have been successfully integrated into a wearable glove [32]. However, normally, they tend to be utilized in a probe-like fashion such as for surgical instruments and struggle to be arrayed [6]. Cirillo et al. presented a photo-diode-based tactile sensor with a 25-element array using photodetectors and one deformable surface [15,33]. However, it requires two proportionally large PCBs to facilitate the readout of the sensing elements and only detects normal force changes [33]. A successful friction-based force and displacement three-axis photodiode-based optical tactile sensor (PapillArray) uses a *camera obscura* and quadrant photodiode to measure the force and displacement of each of nine different sensing units in a 3 × 3 sensing array [16,34], which also requires two PCBs, making the design somewhat bulky and challenging to miniaturize and restricts the sensor’s versatility.

In previous work, we introduced a novel non-camera-based optical-based sensing principle, which uniquely uses the light angle and intensity sensing to infer 3D displacement and 3D force at a point on the outer surface of the sensor [35], and we named the sensing principle *LiVec* as it utilizes light vectors for transduction. However, it requires a proportionally large area for the sensing component (diameter of 8.5 mm). Moreover, the PCB to house the electronic components measured 50 mm by 35 mm in length and width, which makes it difficult to implement on a robotic gripper. While this work successfully allowed us to develop a novel sensing concept with a small overall thickness, our final goal was to cover all parts of a robotic hand with multi-axis distributed sensing, mimicking the human sense of touch.

### 1.2. Current Challenges with Optical Tactile Sensors’ Design

Overall, most existing tactile sensors are too large/bulky to allow their seamless integration onto robotic grippers. Therefore, a big challenge of optical tactile sensors is to reduce their dimensions, and primarily their thickness [9]. Furthermore, the overall sensor, including the processing circuits, needs to come in a compact form factor, fitting the shape of robotic grippers.

Several factors are desired in a tactile sensor to perform dexterous manipulation using a robotic gripper. Firstly, multi-axis force, torque, and deformation sensing is desirable to enable dexterous manipulation. This means a tactile sensor should report forces, torques, and deformations about a contact along/around all 3D spatial axes. The design of current optical tactile sensors shows promise to have six-axis measurements. However, most of them do not seem to have been calibrated for torque, which will affect their ability to select an appropriate grip force when holding an object at a location away from its center of mass. This, in turn, restricts their ability to enable dexterous manipulations of objects [9]. Secondly, the ability to provide multiple localized independent sensing points provides a more-comprehensive measurement of the contact between the sensor and the object, which allows the detection of tactile properties such as contact, slip, pressure, geometry, etc. Lastly, the signal processing requirements of the optical sensing principle should remain simple to allow for fast real-time processing of the information provided by the sensor. The high post-processing requirements of camera-based sensors can potentially hinder the deployment of the sensor [19].

**Figure 1:**
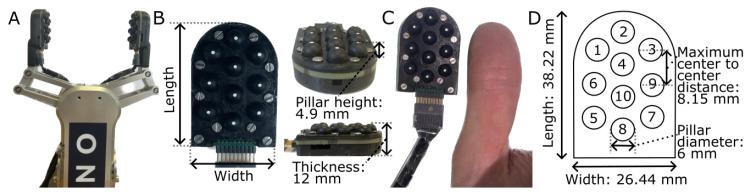
The fabricated LiVec finger. (**A**) Two LiVec fingers mounted on the fingers of an OnRobot RG2 gripper, the gripper the LiVec finger design was targeted at. (**B**) Close-up views of the LiVec finger showing the length (38.22 mm), width (26.44 mm), thickness (12 mm), and sensing unit pillar height from the top plate to the tip of the pillar (4.9 mm) in relation to the sensor. (**C**) A front view showing the overall LiVec finger, illustrating the array of local sensing units next to a human thumb for scale. (**D**) A top view illustration of the LiVec finger, showing the dimensions, length, width, pillar diameter (6 mm), and maximum center-to-center distance (8.5 mm) between the sensing units. The numbers on the top view illustration refer to the local sensing units placements across the sensor.

In this paper, we present the LiVec finger, a novel, small, distributed array of sensing units using the LiVec sensing principle [35] in a fingertip shape (shown in Figure 1), which addresses the shortcomings of the previous version and the current design challenges of optical tactile sensors [35]. The multi-axis distributed LiVec finger is designed to provide localized 3D force and 3D displacement measurements at each local sensing unit and measurements of global forces and torques, all in a compact form factor easily integrated into a commercial robotic gripper. In this paper, we targeted the integration with an OnRobot RG2 two-fingered gripper, as shown in Figure 1A, illustrating that the LiVec finger can fit onto a compact robot finger. Compared to other existing distributed sensing arrays, the optical sensing method used by the LiVec finger enables local sensing units to be arrayed in a manner that is adaptable to the finger area being instrumented. Each sensing unit consists of a simple structure of a soft deformable skin containing a light angle sensor and light emitting diodes (LEDs). Additionally, the design of the LiVec finger brings the major advantage of having a small overall thickness, making it well-suited for commercial grippers, where there may be limited space between the gripper fingers, whereby adding a large-thickness finger would ultimately reduce the stroke length of the gripper finger movement.

### 1.3. Main Contributions of This Work

The main contributions of this work can be summarized as follows: **(1) A novel arrayed design of the LiVec sensing principle:** The design of a non-camera-based distributed optical sensing array. We show that the novel LiVec sensing principle can be arrayed into a distributed tactile sensor. **(2) Characterization of the sensor:** We characterize the LiVec finger, demonstrating its capability for real-time measurement of localized 3D force, 3D displacement, as well as global 3D force and global 3D torque. **(3) Global measurements:** We show that the array of sensing units can estimate the global measurements of force and torque, seldom attempted in the literature. Indeed, the global torque is generally not calibrated for optical sensors. **(4) Compact fingertip form factor:** We show that the LiVec sensing principle can be made into a compact form factor in a fingertip designed specifically for easy integration with a commercially available robotic gripper. **(5) Illustrative demonstration of the sensing capabilities:** We integrated the LiVec finger into a robotic gripper, showing the real-time estimates for a simple manipulation task, highlighting the richness of information available from this sensor, even for rudimentary lift and rotate tasks.

The rest of this paper is organized as follows. In Section 2, we describe the design aims alongside the LiVec sensing principle and fabrication of the LiVec finger. Additionally, the experimental calibration procedure, global force/torque calculation methods, and the demonstration task are presented. Section 3 presents the calibration results, global force/torque results, and then, the demonstration task results. Section 4 compares the LiVec finger against the sensing capability and precision of various state-of-the-art tactile sensors. Section 5 draws conclusions and presents future work.

## 2. Materials and Methods

In this section, we present the design aims and a short description of the working principle of the sensor. We then introduce the LiVec finger design and fabrication, followed by the procedure for calibrating force/displacement measurements for each sensing unit, and then, the global force and torque validation methods. Finally, the methods for the demonstration task are described.

### 2.1. Design and Fabrication

#### 2.1.1. Design Aims

The overall aim of this work was to build a small-thickness, compact, finger-shaped tactile sensing array for the OnRobot RG2 gripper using the LiVec sensing principle. To achieve this, the following underlying design criteria were formulated:

1. An array of sensing units that function independently and, thus, can provide global force/torque information.
2. Have a similar shape to the original OnRobot RG2 gripper end effector shape, for easy integration of the LiVec finger with this target gripper.

Motivated by these design criteria, we present the design of the distributed LiVec finger, which can each estimate the 3D local displacements and 3D local forces and, as one complete sensor, estimate the 3D global forces and torques.

#### 2.1.2. LiVec Finger Sensing Principle

**Figure 2:**
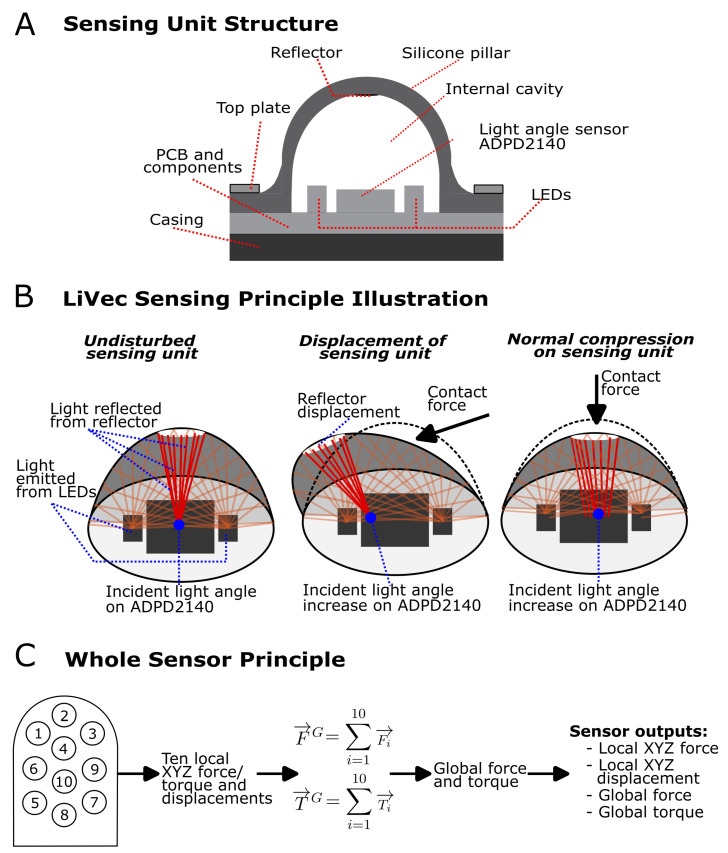
(**A**) A cross-sectional drawing of the a sensing unit of the LiVec finger, showing the components of each sensing unit; PCB, light angle sensor (ADPD2140, Analog Devices, Wilmington, MA, USA [36]), two infrared LEDs, black silicone skin containing the sensing unit protrusion with an internal hollow cavity, white silicone reflector, the top plate used to hold the skin to the PCB, and the case that the sensor sits on. (**B**) An illustration of the LiVec sensing principle for each sensing unit. The undisturbed sensing unit shows the deformable skin in an undeformed neutral position, with a subset of possible light paths from the LEDs illuminating the internal cavity shown. The light that reaches the cavity walls is mostly absorbed by the black silicone skin. The light reaching the reflector is diffusely reflected, and some arrives at the light angle sensor detection area. The average incident angle of the light when the sensor is in a neutral position is 0${}^{\circ }$; however, the light will be incident on all parts of the light angle detection area reflected from all parts of the disk-shaped reflector. The displacement of the sensing unit is shown to illustrate how the average incident angle of the light arriving at the detector changes when the pillar is displaced. The normal compression of the sensing unit causes an increase in the intensity of the light arriving at the detector. This is an illustration, and only some light rays are shown for clarity. (**C**) The LiVec finger’s overall principle is shown in steps: first, from each sensing unit, the 3D force and displacement are obtained, then a sum of the individual forces and torques is performed to obtain the global force and torque experienced by the sensor.

A LiVec finger local sensing unit consists of a deformable, cylindrical pillar-like skin with an internal light reflector embedded at the top of the cavity inside the skin. The skin sits on top of a printed circuit board (PCB) and encompasses the electronic sensing components, which are a light angle sensor (ADPD2140, Infrared Light Angle Sensor, Analog Devices, Wilmington, MA, USA [36]) and two infrared LEDs. The sensing unit layout is shown in Figure 2A. The sensing principle of each local sensing unit is the same as that of the LiVec sensor, described in detail in [35], and an illustration is shown in Figure 2B. However, the sensing unit dimensions presented here are 33% smaller than those presented in [35], and the electronic sensing components are more densely laid out. In particular, the distance between the edge of the light angle sensor and the edge of the LEDs has been reduced.

In summary, each sensing unit infers 3D force and 3D displacement from measurements of light reflected off an internal white reflector embedded at the top of a cavity inside the silicone skin sensing protrusion. Since the 3D position of the internal white reflector is affected by the deformation of the protrusion with the applied external forces, the displacement of, or force applied to, the external tip of the protrusion can, thus, be inferred from the direction and intensity of the light reflected off the internal white reflector. The light angle sensor receives the light coming from the reflector and outputs four photocurrents, which differentially encode the aggregate statistics of the angles of photons arriving at the detector. Additionally, all four photocurrents are proportional to the number of photons arriving at the detector. This allows the displacement and force in the direction normal to the protrusion tip to be inferred using the intensity (sum of the four photocurrents) increase caused by the reflector moving closer to the light angle sensor.

To convert the photocurrents into estimates of 3D force or displacement, a calibration procedure (Section 2.2) and a multivariate polynomial regression were performed. This provides regression equations, which take the four photocurrents and intensity as the inputs and compute the estimates of the XYZ force and XYZ displacement. These regression equations can later be applied to novel photocurrent readings to obtain real-time estimates of force and displacement. Each sensing unit of the LiVec finger uses the transduction method above to obtain local estimates of 3D force and displacement for each sensing unit.

Having estimates of the local forces on each sensing unit, the contribution of that force to the global torques around a chosen origin can be computed using the vector cross-product of the location of the sensing element tip and the force measured at that sensing element. Summing the torque contribution of every sensing element, the global torque is estimated. Similarly, global force is estimated as the vector sum of the forces measured at each individual sensing unit.

#### 2.1.3. Design and Fabrication of the LiVec Finger

The LiVec finger consists of ten compliant sensing units arrayed in a finger-/thumb-like shape, with a compact size of 26.44 mm × 38.22 mm × 12 mm, in width, maximum length, and thickness, respectively (Figure 1C). The ten sensing units are separated by a maximum distance of 8.15 mm from center to center, and the minimum distance is 7.5 mm. The shape was chosen to mimic the human fingertip shape. The sensing units are arranged in three columns: a central column of four sensing units and two side columns containing three units each, allowing for the whole surface of the sensor to be covered. The silicone was dyed black to minimize light reflection from the internal walls of the protrusion cavity, ensuring most of the light arriving at the photodetector comes from the reflector. A silicone skin was chosen so that the finger can conform to the surface and shape of gripped objects where the contact is made, increasing traction.

#### 2.1.4. Electronics

All the electronics of the LiVec finger were assembled on a two-sided, four-layered PCB, with one layer dedicated to the ground and the other layers allowing dense routing of the electronics. The top face of the PCB is populated with only the light angle sensors and LEDs. There are twenty 850 nm-wavelength infrared LEDs (IN-S42CTQIR, Inolux Corporation, Santa Clara, CA, USA) and ten light angle sensors (ADPD2140, Analog Devices, Norwood, MA, USA.) interfaced with five photometric front ends on the underside of the board (ADPD1080, Analog Devices), each ADPD1080 using 14 bit analog-to-digital converters. Each ADPD1080 facilitates the readout of two light angle sensors while simultaneously controlling the pulsing of the LEDs; LED pulsing reduces power consumption and heat generation from the LEDs and enables excellent ambient light rejection. Each ADPD1080 uses an individual GPIO line to control the timing of each sample taken. A 300 mA low-dropout linear regulator (AP22210N-3.3TRG1, Diodes Incorporated, Plano, TX, USA) generates a 1.8 V supply for the ADPD1080s. The LEDs are supplied with 5 V lines, and an IC level shifter (NTS0308EPWJ, NXP Semiconductors, Eindoven, Netherlands.) is used to take the 1.8 V of the GPIOs lines to 3.3 V, the input level of the microcontroller. This enables the readout from the ADPD1080 to a PC via a Teensy 4.1 microcontroller using I^2^C communication.

#### 2.1.5. Mechanical Design and Assembly

**Figure 3:**
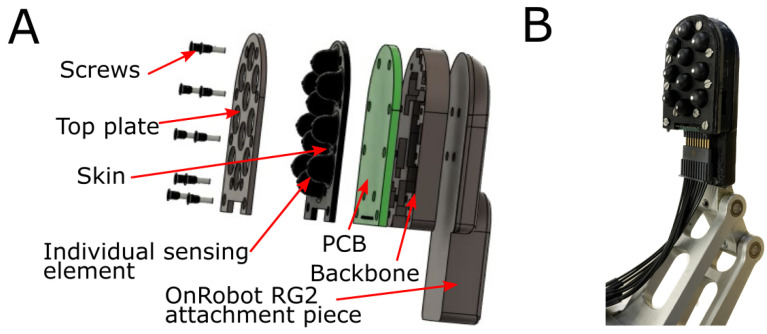
(**A**) An exploded schematic of the LiVec finger, showing the different layers of the sensor. (**B**) An image of the assembled LiVec finger mounted on the RG2 OnRobot gripper finger. The attachment piece for the LiVec replaces the original OnRobot RG2 end effector cap.

The mechanical assembly of the LiVec finger consisted of a top plate, sensor skin, PCB, and sensor backbone (Figure 3A). The top plate, backbone, and mounting piece were 3D printed in black PLA plastic using a Prusa i3 MK3S+ printer (Prusa Research by Josef Prusa). The skin and the top plate were designed to limit optical crosstalk between sensing units by encapsulating each pair of LEDs and associated light angle sensor into the cavity within the sensing unit protrusion. The skin’s individual protrusions have an outer diameter of 6 mm, a height of 5.9 mm from the PCB, and a height at 4.9 mm from the top plate. Each protrusion has an embedded reflector with a diameter of 1.5 mm at the top of a truncated cylindrical cavity at a height of 4.085 mm away from the PCB. The overall sensor has a height of 12 mm from the top of the sensing units’ protrusions to the back of the 3D-printed backbone of the sensor (see Figure 1, which illustrates the height of the sensor). There is an additional detachable mounting piece to mount the array on the OnRobot RG2 gripper finger.

#### 2.1.6. Fabrication of the Sensor Skin

The skin was molded from a two-part platinum cure silicone (Mold Star™ 20T, Smooth-On, Macungie, PA, USA) with a Shore hardness of 20A. The molding process used a top and bottom mold to create the skin, which was 3D-printed using stereolithography ( FormLabs Form 2 printer, using Clear v4 resin, FormLabs, Boston, MA, USA), then rinsed with isopropyl alcohol for 25 min and post-cured at 60 °C for 15 min. The lower mold formed the external protrusion shapes, and the upper mold formed the internal cavities of the protrusions. The skin was then produced in two steps. First, the ten protrusions were molded in a black-dyed silicone using a two-part silicone in a 1:1 ratio. The silicone was then degassed before pouring into the lower mold; the upper mold was attached, and the silicone mix was left to cure. Once the black silicone was set, the second step was creating the silicone reflectors and tracking dots (used for displacement calibration) molded in white-dyed silicone. The same silicone was used and was carefully added via pipette into the void formed at the bottom of the internal cavity of the protrusion to form the reflector; the black silicone internal cavity was molded with a disk-shaped hollow (0.2 mm deep, diameter 1.5 mm) to receive the white silicone reflector. Similarly, the external calibration tracking dot was formed by adding the white silicone into the void at the external protrusion tip (0.2 mm deep, diameter 0.3 mm). Having the reflector and tracking dot made from the same silicone formed a strong bond with the black silicone, resulting in one piece for the skin.

### 2.2. Local Force and Displacement Calibration Procedure

The raw photocurrent reading obtained from the sensor must be converted into localized forces and displacements, which requires a calibration procedure, described below.

#### 2.2.1. Calibration Experiment Test Platform

**Figure 4:**
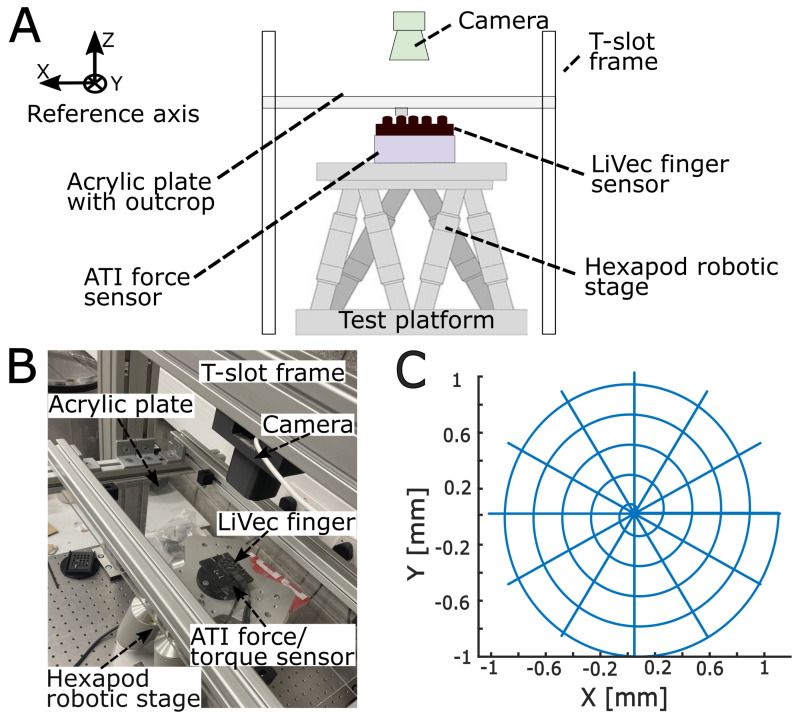
The robotic experimental calibration platform for force and displacement. (**A**) A front-view drawing of the experimental platform illustrating the setup for the calibration experiments. This shows the LiVec finger and ATI Mini 40 force/torque sensor are mounted on the hexapod robotic stage and the T-slot frame around the hexapod, which holds the acrylic plate with the outcrop. The camera is mounted above the hexapod robotic stage and is used as the independent reference of the XY displacement of the sensing unit tip tracking dot. (**B**) An image of the experimental platform setup with each component labeled. (**C**) The calibration patterns. This is the movement shape that the hexapod performs to sample the 3D space of each of the LiVec finger-sensing units. The calibration pattern is formed of a spiral and spoke pattern on the same axis. The patterns were repeated at steps of −0.10 mm of Z compression to a maximum Z compression of −1.50 mm.

The calibration platform consisted of a six-degree-of-freedom (DoF) hexapod robot (H-820, Physik Instrumente, Karlsruhe, Germany), a rigid transparent acrylic plate, either flat or containing an individual small cubic outcrop (also transparent acrylic), a video camera (Logitech Streamcam, Logitech, Lausanne, Switzerland), a six-axis force/torque sensor (ATI Mini 40, ATI Industrial Automation, Dallas, TX, USA), and the LiVec finger (Figure 4). The force/torque sensor and LiVec finger were mounted on the hexapod, via a custom-built 3D-printed support mount. The camera, which was used to image the external tracking dot on each protrusion tip, was mounted approximately 250 mm above the acrylic plate. The camera had a resolution of 1280 × 720 px. The acrylic plate was mounted above the sensor via a T-slot frame. The LiVec finger data were recorded at 390 Hz. The force/torque data were recorded at 200 Hz. The video from the camera was captured at 60 frames per second (FPS). All data were uniformly re-sampled offline to the reference video recording (i.e., resampled to 60 Hz).

#### 2.2.2. Calibration-Data-Collection Protocol

The hexapod was programmed to bring a sensing unit protrusion into contact with the outcrop from the acrylic plate until the desired compression (negative Z displacement) was reached. Then, the hexapod moved laterally (±XY displacement) along one of two different trajectories. The Z displacement of 0 mm was defined manually when the protrusion first made contact with the outcrop, indicated by the increase of force seen in the force/torque sensor measurements. The two lateral movement trajectories (i.e., spiral and spoke patterns; see Figure 4C) were chosen to sample the 3D deformation space of the sensing unit; each pattern was repeated for each sensing unit and for compressions ranging from 0.00 mm to −1.50 mm in −0.10 mm increments. The spiral pattern was coded in polar coordinates with a polar angle that increased linearly with time from 0 to 10π radians (five revolutions) and a radius that increased linearly with time from a radius of 0.00 mm to 1.00 mm at a rate of 0.2 mm per revolution. Additionally, at the start of the spiral pattern, for each sensing units’ compression, data were recorded for zero XY displacement. The spoke pattern had 12 radial lines, each 30${}^{\circ }$ apart, with a length of 1.00 mm. All movement patterns were executed at a speed of 2 mm.s${}^{-1}$. To obtain the actual displacements of the protrusion, the tracking dot on the protrusion tip was used to track the protrusion position throughout the video images. The tracked dot position in pixels was then transformed into millimeters at the level of the acrylic outcrop. The actual deformation of the sensing unit tracking dot was obtained from the difference between the hexapod XY position (after zeroing when contact was first made) and the position of the sensing unit tracking dot; for the most part, the tracking dot did not move, and the hexapod moved the sensor beneath the acrylic plate/outcrop; however, if the sensing unit protrusion slipped, then the tracking dot would move; thus, we corrected for this slipped distance when recording the tracking dot position relative to the sensor/robot frame of reference. The hexapod position was taken for the Z position of the protrusion.

Separate training and validation datasets were acquired following the procedure described above for each of the ten sensing units.

#### 2.2.3. Calibration Procedure

Multivariate polynomial regression was performed for each sensing unit in MATLAB using the function MultiPolyRegress, with equal weight to all inputs and a basic polynomial fit. The targets to the regression were the reference force (from the ATI force/torque sensor) and displacement of the sensing unit tip (from the camera tracking and hexapod position). The inputs to the regression were the four photocurrents of a sensing unit, which were normalized. The normalization step of each unique timestamped photocurrent channel involved calculating an average intensity value (sum of the four photocurrents, i.e., an estimate of the intensity of the light reaching the ADPD2140 light angle sensor) from 200 samples when the sensing unit was undeformed. This value was used to divide (i.e., normalize) all future photocurrent values. As a result, there were five inputs to the regression for each sensing unit, the four normalized photocurrents, and the intensity (i.e., the unnormalized sum of the four photocurrents for that time sample), which are independent variables in the regression model. For each sensing unit, three 4th-order polynomial regressions were performed with the X, Y, and Z displacement as the targets, respectively, and three 3rd-order polynomial regressions were performed with the X, Y, and Z forces as the targets. The model orders were selected through *ad hoc* analysis of model residuals using the training set data only, balancing the improvement of the model fit against the model complexity. Each sensing unit regression model was trained with a minimum, median, and maximum data samples; 58,708, 58,740, and 58,782, due to small variances in the number of samples collected. Each sensing unit regression model was tested with minimum, median, and maximum data samples of 19,901, 20,151, and 20,306, respectively. In detail, one data sample consisted of four normalized photocurrents and the intensity collected from the sensing unit, for that unique time sample, and the whole training dataset consisted of these data samples taken across all the sensing units’ deformable space.

### 2.3. Global Force and Torque Validation Methods

#### 2.3.1. Global Force Calculations

The global force, ${\vec{F}}^{G}=\left(F_{x}^{G},F_{y}^{G},F_{z}^{G}\right)$, was calculated as the sum of the forces measured by each sensing unit (Equation (1)).

(1) $${\vec{F}}^{G}=\sum_{i=1}^{10}{\vec{F}}_{i}$$

where ${\vec{F}}_{i}=\left(F_{xi},F_{yi},F_{zi}\right)$ is the 3D force measured by sensing unit *i*.

#### 2.3.2. Calculating Global Torque

**Figure 5:**
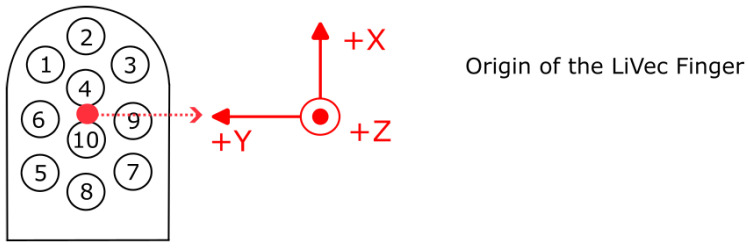
Definition of the LiVec finger’s measurement axes’ directions for all force and torque measurements, where the origin of the frame of reference for each measurement changes depending on whether it relates to a local measurement by a sensing unit or a global measurement obtained by combining all sensing units’ measurements. The origin of the LiVec finger frame of reference used for global force and torque measurements. This is taken as the center of the contact area of the sensing units, with the zero Z position being the top of the sensing units. The center of rotation coincident with the origin of LiVec finger in the sensor frame of reference, which remained fixed at the point illustrated. The numbers on the LiVec finger drawing refer to the local sensing units placements across the sensor.

The origin of the LiVec finger frame of reference is defined as the center of the contact area (center of the sensing units) with the (0, 0, 0) coordinates, taken as here, and the zero Z, taken as the tip of the undeformed sensing units; see Figure 5 This origin of the sensor frame of reference remained fixed in the sensor frame.

**Figure 6:**
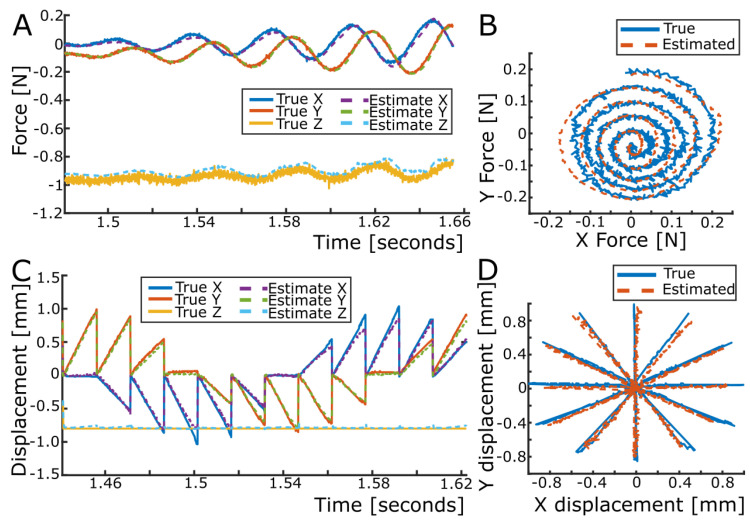
Force and displacement mapping at Z = −0.40 mm compression for sensing unit 1. (**A**) True (solid) and estimated (dashed) local XYZ force for a spiral pattern. (**B**) True and estimated local XY force of sensing unit 1, respectively, for this illustrative spiral pattern. (**C**) True (solid) and estimated (dashed) local XYZ displacement of a spoke pattern. (**D**) True and estimated local XY displacement of sensing unit 1, respectively, for this illustrative spoke pattern.

The global torque, $\vec{T^{G}}=\left(T_{x}^{G},T_{y}^{G},T_{z}^{G}\right)$, was computed relative to the origin of the frame of reference presented in Figure 6B; we selected this origin, for the ease the interpretation of results, to be the center of rotation. The torque produced by each sensing unit was calculated using Equation (2).

(2) $${\vec{T}}_{i}={\vec{r}}_{i}\times {\vec{F}}_{i}$$

(3) $${\vec{r}}_{i}={\vec{l}}_{i}+{\vec{d}}_{i}$$

where, again, ${\vec{F}}_{i}=\left(F_{xi},F_{yi},F_{zi}\right)$ is the 3D force measured by sensing unit *i* and ${\vec{r}}_{i}$ is a vector containing the X, Y, and Z distance from the origin of the frame of reference of the LiVec finger to the tracking dot on the tip of sensing unit *i*. The full definition of ${\vec{r}}_{i}$ is given in Equation (3), where ${\vec{l}}_{i}$ is a vector containing the undeformed coordinates of the sensing unit tracking dot in the sensor frame for reference and ${\vec{d}}_{i}$ is a vector containing the estimation of the 3D displacement of the tracking dot in the frame of reference of sensing unit *i*.

The global torque was, thus, calculated as the vector sum of the torque contribution from each of the ten sensing units, as expressed in Equation (4):

(4) $${\vec{T}}^{G}=\sum_{i=1}^{10}{\vec{T}}_{i}$$

#### 2.3.3. Experimental Global and Torque Validation Protocol

To validate the global forces and torques computed from the forces measured by each sensing unit, we used the same data-collection platform (Figure 4) as in Section 2.2.1. However, for these tests, the acrylic plate was changed to a flat plate. This allowed the comparison of the global force and torque values of the LiVec finger, with the force/torque sensor values taken as the ground truth for both validations. The hexapod was programmed to bring the LiVec finger into contact with the flat acrylic plate (all the sensing units in contact) to the desired compression.

For the global force validation, the hexapod performed both spiral and spoke patterns, similar to the calibration procedure. This was repeated at each compression going from 0 mm to −1.5 mm in −0.2 mm increments.

For the global torque validation, the hexapod moved rotationally around each axis. Rotational movement around the Z axis allowed $T_{z}^{G}$ to be evaluated; similarly, rotational movement around the X axis gave $T_{x}^{G}$, and rotational movement around the Y axis gave $T_{y}^{G}$. The rotation performed around the Z axis was 20${}^{\circ }$ at a speed of 2 mm.s${}^{-1}$, during which all ten sensing units of the LiVec finger slipped. The rotations of 6${}^{\circ }$ at a speed of 2.00 mm.s${}^{-1}$ were performed around the X and Y axes. Each rotational movement for each axis was repeated for compressions going from 0 mm to −1.5 mm in −0.1 mm increments. The LiVec finger sensor unit 3D force values were recorded, from which the LiVec finger global torque values were calculated. The reference force/torque sensor values were also recorded and taken as the ground truth against which to compare the LiVec-finger-calculated global torque values.

### 2.4. Integration of the LiVec Finger with the OnRobot RG2 Gripper: Experimental Demonstration

The LiVec finger was integrated onto an OnRobot RG2 parallel-fingered gripper using an additional mounting piece (Figure 3), which slides onto the metal bone of the gripper finger and was secured with a screw. This was the same method of affixation as the original finger end cap that comes with the gripper. Overall, this means there was no adaption of the gripper to allow the LiVec finger to be mounted, shown in Figure 3B. In order to have real-time estimates, the calibration equations previously obtained were applied to novel normalized photocurrents and the intensity.

#### Experimental Robotic Setup and Demonstration Tasks

The gripper was mounted onto a Universal Robots UR5e robotic arm. The sensor was set to record data during a movement, and a camera (Intel RealSense Depth Camera D455, Intel, Santa Clara, CA, USA) also recorded the movement for visualization purposes. The UR5e arm was controlled using the provided Human Machine Interface (HMI) software (3PE, Universal Robots A/S, Odense, Denmark). Two objects were manipulated during the demonstration: a rigid cuboid box (214.4 g) and a round roll of tightly compacted compressible paper towels (124.3 g).

To show the LiVec finger sensing abilities, the following task was performed as it showed torques changing with movement and showed the response of the sensor when a movement or collision occurred. The gripper grasped the object, lifted the object 10 cm vertically, and rotated it around the sensor’s X-axis (which was kept horizontal in the world frame of reference). Then, the object was physically tapped three times on its top, before being placed back down on the tabletop and released. This was repeated for the second object.

## 3. Results

The calibration results showed that the LiVec finger can successfully estimate local XYZ force and displacement for all ten local sensing units and, thus, simultaneously estimate global force and torque vectors.

### 3.1. Sensing Unit 3D Force and 3D Displacement Validation

Following the calibration procedure of each sensing unit, the obtained regression equations were validated using a test dataset. The forces estimated for each sensing unit using this dataset accurately followed the forces measured by the reference force/torque sensor (see the example for sensing unit 1 in Figure 6A,B). Similarly, the displacement estimates for each sensing unit very accurately matched the reference displacements obtained using the hexapod and the camera (see Figure 6C,D).

**Figure 7:**
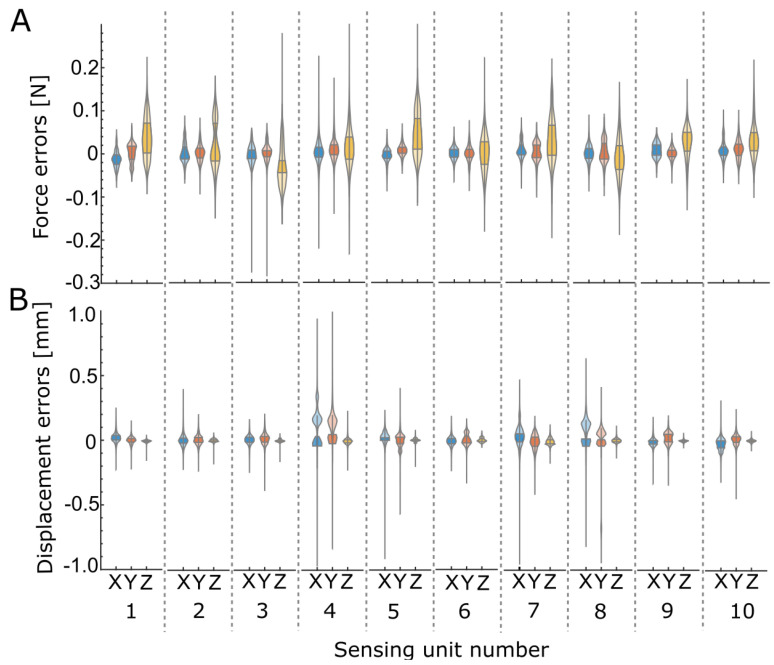
The X, Y, and Z force and displacement error distribution for each of the ten sensing units, visualized in violin plots. Blue represents X, red Y, and yellow Z. The larger shaded area represents the interquartile range (IQR) of the error distribution. (**A**) Force estimate errors. (**B**) Local displacement estimate errors.

The average (across all ten sensing units) force and displacement error statistics are summarized in Table 1, and the full table for each local sensing unit is provided in Appendix A. The precision of the measurement estimates was taken as the standard deviation (SD) of the error, and the bias of the measurement estimates was taken as the mean difference between the measurement estimate and its reference value. The force bias and precision across all sensing units was −2.19 ± 20.89 mN (mean ± SD), 0.89 ± 19.19 mN, and 12.20 ± 43.22 mN for the X, Y, and Z forces, respectively. The displacement bias and precision across all sensing units were 2.38 ± 56.70 μm, 4.73 ± 50.18 μm, and −4.65 ± 13.83 μm in X, Y, and Z, respectively. Each sensing unit of the LiVec finger had a tested deformable range of ±2.00 mm in X and Y and −1.50 mm in the Z direction. Figure 7 shows the force and displacement error distributions for each sensing unit visualized in violin plots, showing that the errors across the local forces and displacements were centered around or near zero and tended to be relatively symmetric.

### 3.2. Global Force and Torque

**Figure 8:**
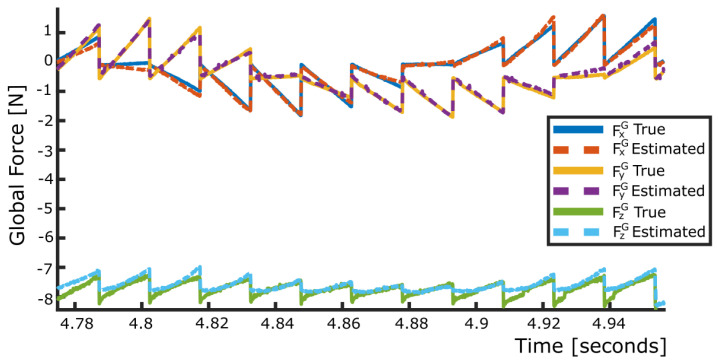
Global force mapping at −0.80 compression. True (solid) and estimated (dashed) global XYZ force for a spoke pattern, validating the calculation of the global force for the LiVec finger.

To validate the global force and torque measurements obtained with the LiVec finger, all protrusions were brought into contact against the acrylic plate and moved laterally or rotationally. Figure 8 shows the X, Y, and Z forces measured by the force/torque sensor values against $F_{x}^{G}$, $F_{y}^{G}$, and $F_{z}^{G}$ for one compression in a spiral pattern of the global force validation tests. This highlights the accuracy of computing $F_{x}^{G}$, $F_{y}^{G}$, and $F_{z}^{G}$ as the sum of the X, Y, and Z forces of all local sensing units. The $F_{x}^{G}$, $F_{y}^{G}$, and $F_{z}^{G}$ bias and precision (mean ± SD) were 19.60 ± 111.61 mN, 7.60 ± 91.83 mN, and −54.51 ± 139.10 mN, respectively, over a tested force range of ±2.00 N for the X and Y axes and 0.00 N to −12.00 N for the Z axis; this is also summarized in Table 2.

**Figure 9:**
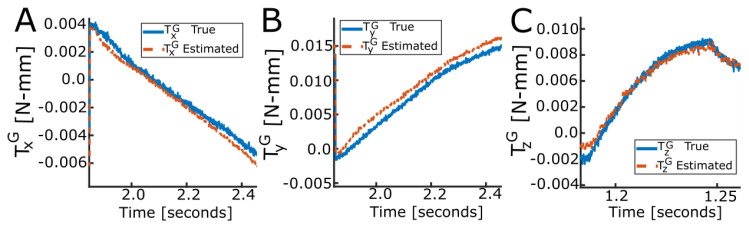
Example of global torque estimation. True (solid) and estimated (dashed) global XYZ torque for rotational movements. (**A**) X axis torque validation at Z = −0.50 mm compression for a negative 3${}^{\circ }$ rotational movement. (**B**) Y axis torque validation mapping for Z = −0.50 mm compression for a positive 3${}^{\circ }$ rotational movement. (**C**) Z axis torque validation mapping for Z = −0.50 mm compression for a positive 10${}^{\circ }$ rotational movement.

Figure 9 shows the X, Y, and Z torques measured by the ATI force/torque sensor against $T_{x}^{G}$, $T_{y}^{G}$, and $T_{z}^{G}$ for separate rotational movements about each axis and at one compression. This illustrates how $T_{x}^{G}$, $T_{y}^{G}$, and $T_{z}^{G}$ accurately matched the torque measured with the reference force/torque sensor, validating the local and global torque calculations. The $T_{x}^{G}$, $T_{y}^{G}$, and $T_{z}^{G}$ bias and precision (mean ± SD) were −0.39 ± 1.90 N-mm, 0.11 ± 1.54 N-mm, and 1.49 ± 1.26 N-mm; this is also summarized in Table 2.

### 3.3. Demonstration Task Results

**Figure 10:**
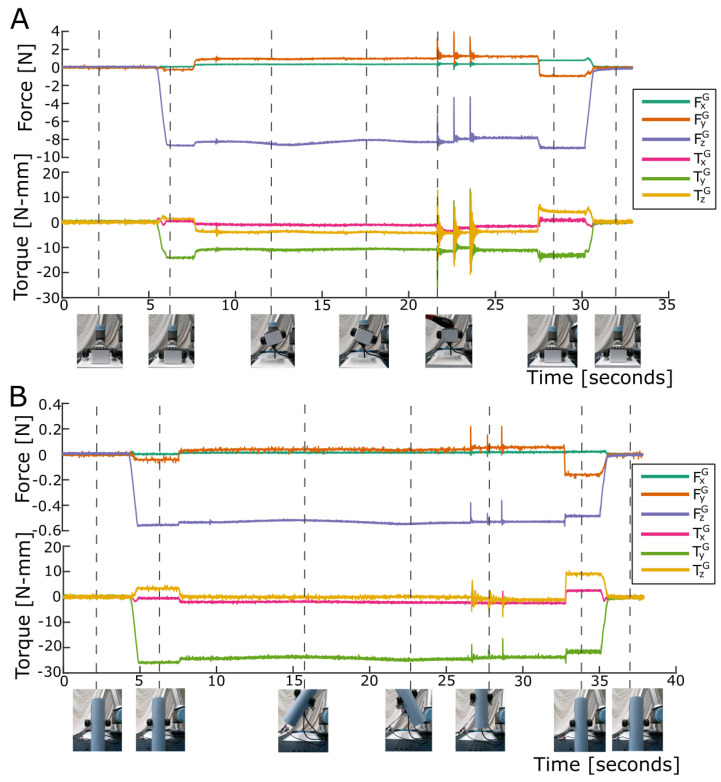
The global forces and torques for the demonstration robotic manipulation task using two objects. $F_{x}^{G}$ is in green, $F_{y}^{G}$ in orange, $F_{z}^{G}$ in purple, $T_{x}^{G}$ in pink, $T_{y}^{G}$ in green, and $T_{z}^{G}$ in yellow. The images are frame captures from the video at the time indicated. (**A**) The cuboid box manipulation. (**B**) The roll of paper towels manipulation.

The LiVec finger successfully calculated and transmitted (for logging) real-time data when deployed in the robotic setup. The global force and torque output of the LiVec finger can be seen in Figure 10A when lifting the cuboid box and Figure 10B for the roll of paper towels, which was lifted off-center. Initially, the LiVec finger was not grasping anything, so all channels read close to zero. When the gripper grasped the box, measurements on all channels changed, with a distinctive decrease in the Z force and displacement, clearly indicating that contact with the object was made and that the sensing units were experiencing compression as the gripper tightened its grasp. Later, when the box rotated around the (horizontal) X axis, changes in the torque measurements can clearly be seen as the share of the object’s weight being carried by each of the gripper fingers changing. Then, later, at 21.4 s, 22.3 s, and 23.6 s, we tapped the box, and this caused a visible vibration each time, with the box moving slightly due to slippage. Lastly, when the box was released, the sensor outputs returned to zero. For the cuboid box, all the sensing units made contact with the object; however, when grasping the roll of paper towels, not all the sensing units were fully in contact, and it was grasped off-center along the X axis. This highlights that the sensor can work with a range of objects and provides important information about the contact between the sensor and the object.

**Figure 11:**
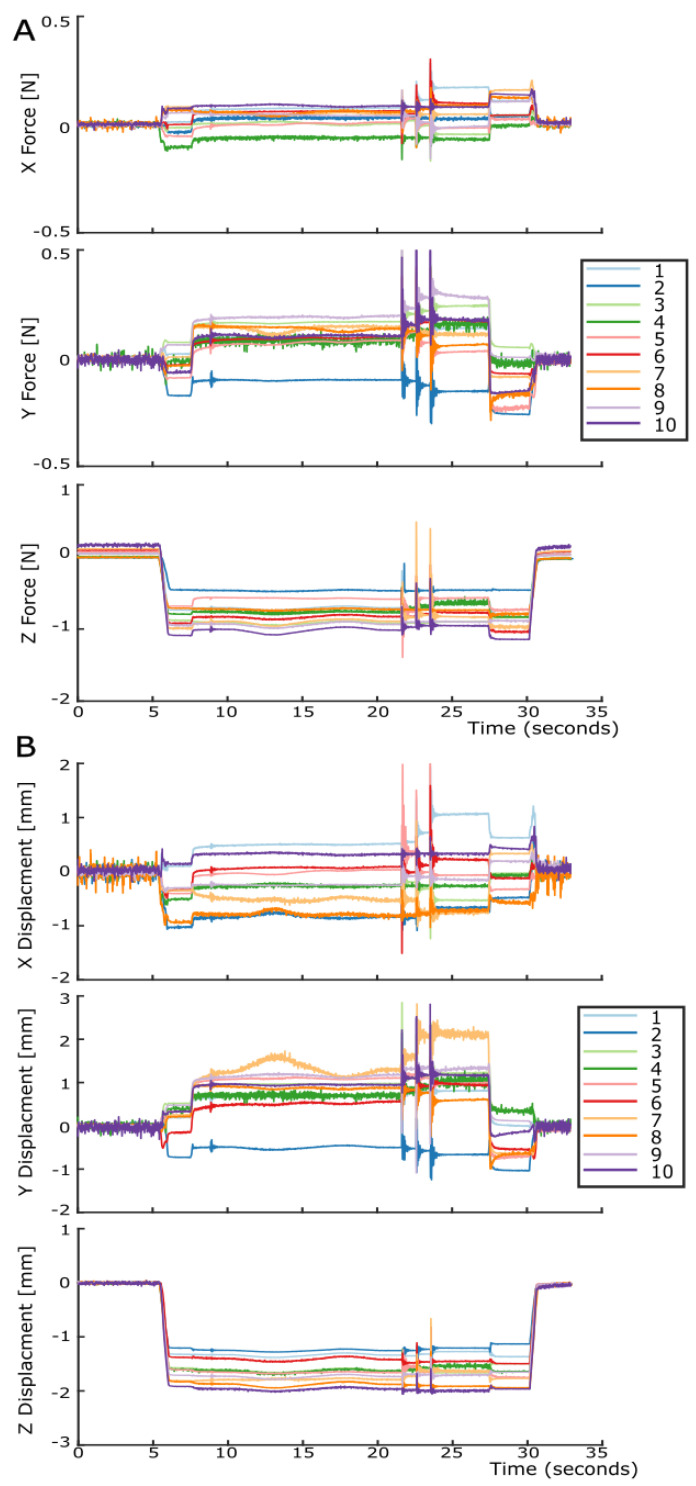
Illustration of all sensing units’ outputs during the robotic manipulation of the cuboid box. For both the XYZ force and XYZ displacement graphs, with sensing units numbered 1 to 10. (**A**) The local XYZ force for each sensing unit with all X forces, Y forces, and Z forces in separate graphs. (**B**) The local XYZ displacement for each sensing unit, X displacements, Y displacements, and Z displacements in separate graphs.

Figure 11A shows the XYZ forces for all ten sensing units, and Figure 11B shows the XYZ displacements for all ten sensing units for the manipulation of the cuboid box. Similarly, as with the global forces/torques, zero readings when the cuboid cox was not grasped can be seen, followed by changes on all axes for both force and displacement when the cuboid box was grasped. Again, when the box was tapped, you can see the clear vibration in all sensing units channels in response to these events. Lastly, all sensing units outputs returned to zero when the box was released. Figure 11 highlights that each sensing unit’s estimates for both force and displacement were individual. It is important to note how the outputs of sensing unit 4 were much nosier than the other sensing units; from our investigations, we determined that the routing in the PCB caused this, possibly as a result of the close proximity of digital and analog lines, and future work will resolve this.

The integration of the LiVec finger on the gripper and the results of the object manipulations showed that the sensor can provide real-time estimates in a real-world use case. The LiVec finger’s sensing capabilities are also highlighted in Supplementary Video S1.

## 4. Discussion

This paper introduced a novel, distributed array of sensing units using the LiVec sensing principle [35] in a fingertip shape (LiVec finger). The LiVec finger can estimate XYZ force and torques and XYZ displacement at 10 locations on its skin, alongside global XYZ force and torques. The sensor size is similar to that of a human thumb and designed in a fingertip shape, making it suitable for use on robotic fingers without the need to adapt the robotic finger. The LiVec finger demonstrated that the LiVec sensing principle can be arrayed into a distributed sensor and can measure the six-axis global force/torque in the sensor frame of reference with excellent precision (Table 1), rivaling other multi-axis state-of-the-art sensors. Table 3 compares the multi-axis force/torque sensors’ characteristics and, in particular, the precision of the sensors, highlighting the relative precision of the LiVec finger. We also demonstrated the utility of the LiVec finger during a robotic grasping task.

The key specifications of a tactile sensor will differ depending on the intended application. For example, GelSight sensors [11,38] have especially good spatial resolution and are generally used for identifying defects in surfaces. Another example is the PapillArray sensor, which is designed to detect incipient slip for grip security, and as a result, it can successfully maintain grasps using this method in real-time despite having a relatively low spatial resolution [37]. The LiVec finger was designed with the proposed aim of improving robotic dexterous manipulation. To do so, the LiVec finger was designed to provide distributed multi-axis force and deformation sensing and to provide distributed and six-axis information about the contact between the robot finger and object. To facilitate its integration into an existing robotic gripper, the LiVec finger was designed in a fingertip shape particularly suited to the OnRobot RG2 parallel-fingered gripper. If the only aim of the LiVec finger was to have a small thickness, then Zhou et al.’s optical fiber sensor would be a better approach as it has a 75% smaller thickness [31] than the LiVec finger. However, Zhou et al.’s design has a three-times worse force precision in X and Y and only has one sensing unit [31], compared with the ten sensing units of the LiVec finger, making it less useful for detecting slip events or estimating object shape, for example.

### 4.1. Advantages of the Sensor Design

The distributed nature of the LiVec finger comes from the separate sensing units of the array, which are fully independent of each other, hence eliminating mechanical and optical cross-talk between sensing units. Combining the information from all sensing units enables the LiVec finger to infer the information at a global level.

The idea of having a distributed array of independent pillars, using an optical sensing principle capable of sensing both force and displacement was proposed by Khamis et al. in [34], for the purpose of incipient slip detection, as some sensing elements slipping before gross slip occurs provides an early warning system for grip force correction [34]. The LiVec finger builds on the PapillArray sensor concept, having multiple independently moving sensing units using an optical transduction method [16]. However, the LiVec finger uses a more-sophisticated sensing principle, specifically using a light angle sensor for sensing, eliminating the need to have a pinhole to create a light spot, which enables the sensing. The transition to light angle sensing to measure sensing element deformation means that our sensor can be manufactured using only one PCB, thus reducing the number of manufacturing steps and, most importantly, reducing the thickness of the sensor. Furthermore, we now have the potential to manufacture using flexible PCB technology, to add tactile sensation of non-planar surfaces.

Having each sensing unit able to detect 3D force and local slip using the local 3D displacements means that both the normal and tangential forces are known locally across the sensor, as well as when the instant incipient slip begins occurring; from this, the friction coefficient can be computed, and as a result, the grip force can correctly be adjusted. Grip force adjustment was shown to be successful using the PapillArray sensor [37]; we expect that this could be achievable using the LiVec finger. Another benefit of having independent sensing units is in interacting with objects of varying sizes and shapes, as some sensing units can be in contact with the object while others are not. This is different from camera-based optical tactile sensors [10,27], in which the skin design typically leads to the movement of one part of the skin to be coupled to movement in another part of the skin. Furthermore, some camera-based sensors may struggle to determine whether part of the sensor skin is in contact with the object at all; the GelSight sensor, which is only coated with a thin layer of paint, can achieve contact area estimation, while the TacTip has a much thicker outer skin, making the contact areamore difficult to determine.

Another advantageous property of the LiVec finger is its small thickness. The LiVec finger possesses a smaller thickness than other tactile sensor, specifically camera-based tactile sensors, such as the compact GelSight sensor built for the Baxter Robot gripper with a thickness of 60 mm in a cuboid shape [11], the GelSlim finger, which is twice as thick [27], the GelSight Wedge, which is comparable in length and dimension to the human finger, but requires a triangle-like depth to allow the sensing [12], and the TacTip, the 2021 version of which has a body depth of 45 mm [10]. The uSkin sensor [21] showcases a smaller thickness (5.85 mm), but uses a magnetic sensing method; magnetic methods are prone to (ferro)magnetic interference, which may limit their utility in industrial applications. The LiVec finger’s thickness is also comparable to non-camera-based optical tactile sensors, such as Cirillo et al.’s dense optoelectronic sensor array with 25 photodiodeswith a 21 × 21 mm${}^{2}$ sensitive area and a 10 mm deformable layer height [33]. However, it requires two stacked proportionally large PCBs compared to the sensitive area covered by a case (90 mm × 21 mm), which is over four-times larger than the sensitive area. This results in a proportionally large device, with the two PCBs increasing the overall dimensions [33]. The PapillArray sensors also do not use a camera, reducing their thickness, but similarly require two PCBs (or at least a quadrant photodiode positioned beneath a pinhole) to implement the sensing principle, which increases the sensor’s overall thickness [34]. Initially, the sensor had an arbitrary deformable pillar height of 20 mm in [16], and more recently, the commercially available PapillArray sensor from the company Contactile has an overall height of 12.8 mm, with 2/3 of the height coming from the casing [20].

The small size and distributed nature of the LiVec finger make it unique in the way it can detect forces/torques, making it much more applicable and adaptable to tactile sensation for robotic grippers. This is in contrast to commercial force/torque sensors, which are rigid and are not finger-shaped; they are typically intended to couple mechanical parts to each other while measuring the forces and torques exchanged between these parts. The smallest ATI Industrial Automation force/torque is a Nano-17, which is 17 mm in diameter and has a 14.5 mm height [22]; this is thicker than our sensor and gives a single point measurement. Furthermore, these devices are extremely expensive, at 5k–10k USD per sensor.

Our optical transduction method does not require a high computational power to process the raw sensor data. This is an advantage when compared to camera-based tactile sensors that require significant computation to extract the forces and deformations of their skins from high-resolution video. One method to reduce this high processing power requirement for camera-based sensors is to have event-based cameras, which only record data when a significant change occurs on a given pixel [41]. But, this means that static forces cannot reliably be determined, as the integration of transient events will include an accumulating integration noise; however, detecting both static and dynamic forces has been identified as a minimum functional requirement for mimicking human in-hand tactile information [2]. This highlights the usability and functionality of the LiVec finger, as it does not need to overcome these challenges.

### 4.2. Limitations of the Sensor

Of course, the proposed design is not without its limitations. This LiVec finger was made with a rigid PCB, restricting it to only being applicable for flat robotic fingers. This imposes a constraint on the applications in which the LiVec finger can be used, as the sensor only has sensing units on one side. It would be desirable to have the sensor cover the surface of a curved robotic finger. This would help ensure that the contact between sensing units is roughly parallel to the object surfaces no matter what the object shape encountered. This would allow our sensor to conform to non-flat surfaces while providing a larger overall sensing area, hence making the sensor more versatile [4,9,23]. A curved sensor would open new possibilities in terms of applications, such as collision detection and object exploration [4]. This means that the next version should be able to cover curved surfaces by utilizing flexible PCB technology.

In this design, it was decided that the local sensing units should have the same height, as this met the overall design aims. Crucially, this LiVec finger was developed to explore the feasibility of arraying the sensing principle of the original LiVec sensor design [35], in particular if it was possible in terms of the electronic routing and miniaturization of the sensing unit size. However, if we wanted to better detect incipient slip, it would be more appropriate to design the sensing units heights to be different, as this would promote incipient slip on the smaller protrusions (usually arranged around the periphery of the array) and help in the prevention of gross slip [9].

One limitation of our optical transduction method is that is can be affected by the external environment. External temperature change can affect the sensors output, as it causes an electronic drift and/or the silicone skin’s expansion. To combat this issue in a similar way to other commercial sensors, such as the ATI Industrial Automation force/torque sensors [42], the sensors need biasing before use to correct estimates for thermal drift [35].

External optical interference also has the potential to affect the sensor estimates. To reduce this occurrence, the sensor uses infrared LEDs and a visible light blocker on the light angle sensor [36]. Additionally, the silicone sensor skin was dyed black to reduce external and internal optical inference within the sensing principle. Finally, the ADPD1080 photometric front-end amplifiers and data acquisition units pulse the LEDs with a very short duty cycle and perform band-pass filtering to reject ambient light.

A current restriction of our novel sensing principle, when instantiated in an arrayed design, is that each sensing unit needs a separate internal cavity. Moreover, the skin must be held in place using a top plate to prevent blistering of the skin from the PCB between the sensing units. If blistering occurs, it could result in light contamination between the local sensing units, which could influence the measured forces and displacements. The top plate is used to prevent this, but this prevents the sensor from having a contiguous elastomer skin; gluing or another method of attaching the skin may be a viable alternative.

### 4.3. Potential Applications of the LiVec Finger

Having a six-axis force/torque sensing array, in particular, is a valuable tool for robotic gripping, as it provides crucial information about the object–end effector interface [9,19]. Having localized force and displacement measurements is particularly useful for incipient slip detection, as it can detect when a gripper loses a stable grasp of the object. Knowing all of this would allow robotic grippers to perform precise and adaptable tasks while ensuring the stability of the grasp [43]. More-advanced algorithms have allowed adaptive grasping, slip prevention [43,44], exploration, object handling, and grasping of unknown objects in unstructured environments [45]. Wang et al. added a fingertip force/torque sensor to a two-finger gripper to identify the parameters needed for stable grasping, illustrating the need for torque measurements [46]. Similarly, Mu et al. provided the i-Limb robotic hand with distributed force sensing using flexible sensors on the fingers and thumb. They performed standard grasping modes and analyzed the contact forces. The contact forces clearly demonstrate that multi-axis force information is needed to understand the object/finger interface and allow a clear distinction between object grasps [47]. Using a combination of the uSkin sensor, a 3-axis force sensor, and a 6-axis force/torque sensor on the Allegro hand, Funabashi et al. showed that they could successfully provide stable in-grasp manipulation once the tactile force information was available and processed [48]. As the LiVec finger provides distributed three-axis force and displacement measurements at 10 local locations, as well as the global 3D force and torque, we expect the sensor has the ability to improve dexterous robotic manipulations.

In most robotic grippers, torque is most commonly measured uniaxially at the joints of the gripper, enabling more-controlled movements of the gripper, but lacking any torque information at the object–gripper interface [49]. However, having torque information at the object–gripper interface allows more tactile information to be extracted, such as determining the center-of-pressure estimation of an object, which is useful for grip force adjustment and ensuring object stability [49,50]. This is similar to humans: when controlling grip force, we take into account force and torque information, and if the torque is excessive, we will adjust our grip on the object by re-grasping closer to the center-of-mass [51]. Performing this sensing at the fingertips of the gripper removes the complexity of having to integrate additional force/torque sensors into the linkages and joints of the gripper itself. In robotic grippers, Feng et al. were able to implement center-of-mass-based re-grasping using visual tactile sensors and torque sensors [52]. Here, the torque sensors were essential for determining slip and adjusting the grasp [52]. The ability to measure torques at the object–gripper interface enables precise grip force adjustments and opens up new uses of tactile sensors, such as being used for improving the grasping abilities, for example, in surgical tools [53,54] and precise positioning and applying rotation forces in assembly tasks [50].

## 5. Conclusions

In this work, we proposed a small-thickness distributed optical tactile sensor, designed in a finger shape, to improve robotic dexterous manipulation. The distributed array of sensing units was based on our novel instrumentation approach (LiVec [35]), allowing the precise estimation of local XYZ force and displacement at ten locations, while providing global XYZ force and torque measurements. These properties will facilitate tactile sensing in various applications, primarily using displacement and force information to control and monitor tasks requiring responsiveness and in-hand dexterity. The main contributions of this work are:

1. First, a tactile sensor array using the LiVec sensing principle;
2. The characterization of six-axis global force/torque measurement using a tactile array;
3. The validation of global force and torque estimates;
4. The demonstration of real-time sensor estimates, including torque, for a simple robotic manipulation example.

The LiVec finger’s compact size, similar to that of a human thumb, and its small thickness make it suitable for integration into existing robotic fingers without requiring extensive adaptation. The distributed array of sensing units may give it the potential to provide early warnings of impending gross slip and triggering grip force adjustment, enhancing its utility in object manipulation. The LiVec finger’s potential applications are vast, ranging from improved robotic gripping and manipulation, to prosthetic devices, where it could offer precise tactile feedback and enhance grasping capabilities.

In future work, we will explore the use of this sensor in more-complex gripping applications, further showing the utility of the sensor and its capacity to detect incipient slip and adjust the grip force accordingly. Furthermore, variations in the heights of local sensing units could enhance its slip detection capabilities, along with exploring the vibration-sensing abilities of the distributed array. While this LiVec finger design shows substantial promise, a restriction of the sensor is that it is limited to flat robotic fingers. Our future work will involve iterations that explore more-versatile designs that cover curved surfaces using flexible PCBs, providing a larger sensing area.

## 6. Patents

S.J. Redmond, D. Córdova Bulens, O. Leslie, P. Martinez Ulloa. Optical-based Tactile Sensor. U.K. Patent Application No. 2207451.2.

## Figures and Tables

**Table 1:** Summary of the bias and precision of force and displacement estimates of each of the ten sensing units. The overall average bias (mean of ten bias values) of the sensing units and the average precision (±SD, as the mean of ten precisions) for both the X, Y, and Z force (mN) and X, Y, and Z displacement (μm).

	Sensor Unit Force		Sensor Unit Displacement	
Axis	Bias (mN)	Precision (mN)	Bias (μm)	Precision (μm)
X	−2.19	20.89	2.38	56.70
Y	0.89	19.19	4.73	50.18
Z	12.20	43.22	−4.65	13.83

**Table 2:** Global force/torque estimate error table: the bias (mean) and precision (SD) for the X, Y, and Z global force/torque.

	Force		Torque	
Axis	Bias (mN)	Precision (mN)	Bias (N-mm)	Precision (N-mm)
X	19.60	111.61	−0.39	1.90
Y	7.60	91.83	0.11	1.54
Z	−54.51	139.10	1.49	1.26

**Table 3:** Comparison of the characteristics of the multi-axis force-based tactile sensors. The sensor size refers to the the whole sensor including the sensor casing, where L is the length, W is the width, and H is the thickness. Not applicable is referred to as N/A. Information not available is defined as —. Force/torque is defined as F/T.

Sensor	1. LiVec Finger	2. Contactile PapillArray	3. GelSight	4.	5.	6. uSkin	7.	8.
**Transduction principle**	Optical (photodiode-based)	Optical (photodiode-based)	Optical (camera-based)	Optical (optical fiber)	Capacitive	Magnetic	Piezo-resistive	Resistive (strain gauges)
**Measurement**	6-axis F/T	3-axis force	3-axis force	3-axis force	3-axis force	3-axis force	6-axis F/T	3-axis force
**Force sensing precision (mN/ N-mm)**	Fx: 21 Fy: 19 Fz: 43 Tx: 1.9 Ty: 1.54 Tz: 1.26	Fxy: ±50 Fz: ±50	Fz: 50	Fxy: 81.1 * Fz: 28 *	Fx: 0.82 Fy: 0.54 Fz: 0.10	—	Fx: 919 Fy: 956 Fz: 995 Tx: 0.680 Ty: 0.543 Tz: 0.785	Fx: 30 Fy: 30 Fz: 10
**Number of sensitive elements**	10	9	—	1	1	25	5	16
Overall sensor size (L × W × H) (mm) **	26.4 × 38.2 × 12.0	24.0 × 30.6 × 12.6 ***	35.0 × 35.0 × 60.0	23.0 × 23.0 × 3.0	2.5 × 2.5× 0.66 ${}^{†}$	35.0 × 30.0 × 28.0	10.0 × 10.0 × 1.3 ${}^{†}$	110.0 × 54.0 × 30.0
**Shape of sensor**	Fingertip	Square	Square	Fingertip	Square	Fingertip	Square	Square
**Robotic gripper integration**	OnRobot RG2 gripper	2F-140, Robotiq	Modified Batex robot	2F-85, Robotiq Inc. (Levis, QC, Canada)	N/A	3D-printed fingertip for the Allegro Hand	N/A	Custom 3D-printed parallel gripper
**Publication year**	2023	2021	2014	2022	2017	2018	2020	2023
**Reference**	${}^{††}$	[20,37]	[11,38]	[31]	[39]	[17]	[40]	[14]

^*^ These values correspond to force sensitivity, where sensitivity is in counts/mN; ^**^ the size refers to the sensor presented in the reference in the row; ^***^ the thickness stated here is the combined casing and pillar height; ^†^ no casing is presented, so not included in the size noted here; ^††^ our sensor presented here.

## Data Availability

The data presented in this study are available upon request from the corresponding author, with videos of particular interest available in the Supplementary Material.

## Supplementary Materials

The following Supporting Information can be downloaded at: https://www.mdpi.com/article/10.3390/s23249640/s1. Video S1: Real-time demonstration of LiVec finger’s sensing capabilities.

## Abbreviations

The following abbreviations are used in this manuscript:

MEMS	Microelectromechanical system
PCB	Printed circuit board
LED	Light-emitting diode
GPIO	General-purpose input/output
I^2^C	Inter-integrated circuit
PC	Personal computer
PLA	Polylactic acid
SFI	Science Foundation Ireland
F/T	Force/torque

## Appendix A

**Table A1:** Local sensing units’ estimates error table: the overall bias, precision for both the X, Y, and Z force (mN) and X, Y, and Z displacement (μm).

		Force		Displacement	
		Bias (mN)	Precision (mN)	Bias (μm)	Precision (μm)
Sensing unit 1	X	−7.92	18.60	0.05	37.42
	Y	−9.89	22.62	−2.35	27.78
	Z	38.25	46.67	−8.05	11.83
Sensing unit 2	X	0.10	22.21	−0.08	53.35
	Y	2.27	20.71	8.20	26.10
	Z	19.59	54.99	−3.78	11.34
Sensing unit 3	X	−3.68	19.75	10.54	71.10
	Y	−23.79	52.75	0.86	14.42
	Z	23.79	52.75	0.86	12.41
Sensing unit 4	X	−11.27	23.99	1.01	49.21
	Y	12.87	24.77	26.80	48.48
	Z	3.21	29.00	−8.10	12.41
Sensing unit 5	X	−3.86	14.98	33.34	60.76
	Y	1.3	14.97	12.62	90.80
	Z	37.04	49.61	−4.38	18.43
Sensing unit 6	X	−8.56	20.19	−9.35	47.19
	Y	−13.60	19.05	16.92	45.79
	Z	4.31	32.44	−7.12	15.76
Sensing unit 7	X	8.56	24.23	−0.79	43.98
	Y	9.47	17.44	−22.70	42.79
	Z	28.94	54.34	−7.66	17.97
Sensing unit 8	X	5.72	25.87	−1.43	57.79
	Y	2.13	14.32	−14.90	79.13
	Z	−25.17	36.11	−4.01	14.80
Sensing unit 9	X	−0.20	15.05	0.84	87.14
	Y	−0.54	13.36	−0.37	40.23
	Z	−25.17	44.51	0.76	10.71
Sensing unit 10	X	−1.57	24.07	−10.29	60.31
	Y	11.36	19.73	9.72	44.93
	Z	15.26	31.84	12.10	12.10
